# Extremely high erythrocyte sedimentation rate revisited in rheumatic diseases: a single-center experience

**DOI:** 10.55730/1300-0144.5536

**Published:** 2022-08-30

**Authors:** Zehra ÖZSOY, Emre BİLGİN, Melek Seren AKSUN, İmdat EROĞLU, Umut KALYONCU

**Affiliations:** 1 Division of Rheumatology, Department of Internal Medicine, Faculty of Medicine, Hacettepe University, Ankara, Turkey; 2 Department of Internal Medicine, Faculty of Medicine, Hacettepe University, Ankara, Turkey

**Keywords:** Erythrocyte sedimentation rate, albumin, ferritin, thrombocyte

## Abstract

**Background/aim:**

The objectives were to define the distribution of rheumatic diseases in patients with erythrocyte sedimentation rate (ESR) ≥ 100 mm/h and to find variables that can differentiate main study groups from others.

**Materials and methods:**

Charts of patients admitted with ESR ≥ 100 mm/h between 2015 and 2020 were reviewed. Patients were divided into four diagnostic groups based on etiology: infection (without a rheumatic diagnosis), oncologic (without a rheumatic diagnosis), rheumatic, and no definitive diagnosis. Patients with the rheumatic diagnosis were divided into three main study groups: those who had been recently diagnosed with a rheumatic disease, those who had a flare-up of the rheumatic disease, and those who had an infection in the course of the rheumatic disease. Appropriate statistical tests and decision-tree analysis by R and ROC curve were applied. p < 0.05 was considered statistically significant.

**Results:**

A total of 2442 patients (311 (12.7%) with rheumatic disorders) were identified. Eighty-six (27.7%) patients had newly diagnosed rheumatic disease (41; 47.7% with vasculitis); 111 (35.7%) had rheumatic disease flare-up (92; 82.9% with inflammatory arthritis); and 114 (36.6%) had coexisting infection (61; 53.5% inflammatory arthritis). Irrespective of the study group, the most commonly encountered diseases were rheumatoid arthritis and spondyloarthritis. Serum albumin levels (2.78 mg/dL) and platelet count (290/mm^6^) were valuable to discriminate disease flare-up and coexisting infection; moreover, high ferritin levels were accounted for adult-onset Still disease among patients with newly diagnosed rheumatic diseases.

**Conclusion:**

Extremely high ESR is still a valuable clinical parameter, and rheumatic causes are significant besides malignancy and infections. Albumin, thrombocyte count, and ferritin are other tests that clinicians should consider when caring for a patient with ESR ≥ 100 mm/h who has rheumatic disease.

## 1. Introduction

Red blood cell descendence rate in uncoagulated venous blood in a given period is defined as the erythrocyte sedimentation rate (ESR) [1]. ESR is elevated in acute tissue damage; infectious conditions such as cellulitis, pneumonia, and soft tissue infections; rheumatic diseases such as rheumatoid arthritis, ankylosing spondylitis, vasculitis, systemic lupus erythematosus, connective tissue disorders, and malignancies such as lung cancer and lymphoma, as well as in physiological processes like pregnancy [1]. Clinicians have long used ESR to assess acute phase response. However, it has a low sensitivity and specificity [2]. An ESR of ≥100 mm/h generally indicates the presence of a severe underlying disease.

In Fincher and Page’s study of 1006 patients, most patients with ESR ≥ 100 mm/h had malignancy (33%), rheumatic diseases (17%), and infection (14%) [3]. In another large study of 4807 patients with an ESR ≥ 100 mm/h, 38% of the patients had autoimmune diseases with the distribution as follows: rheumatoid arthritis was the most common, polymyalgia rheumatica, systemic lupus erythematosus, giant cell arteritis, and gout were the other common rheumatic diseases [4]. The reasons for this significant increase in ESR in patients with the rheumatic disease could be due to the rheumatic disease itself, a flare-up of the underlying disease, or infections added to the clinical picture of the rheumatic diseases [5]. Hence, the characteristics of these three conditions are not well documented in the literature.

The objectives of the present study were to determine the distribution of rheumatic diseases in patients with ESR ≥ 100 mm/h and to identify the factor which may aid in discriminating study groups.

## 2. Materials and methods

### 2.1. Patients and study groups

In this descriptive study, charts of patients who applied to Hacettepe University Medical Faculty Hospital with an ESR ≥ 100 mm/h in the laboratory examination between 2015 and 2020 were reviewed. Patients with ESR ≥ 100 mm/h were divided into four diagnostic groups based on etiology: infection (without a known rheumatic diagnosis), oncologic (without a known rheumatic diagnosis), rheumatic, and no definitive diagnosis. The current study included patients with a definite rheumatic disease with an ESR of ≥100 mm/h. Among patients with rheumatic disorders, patients with missing demographic or laboratory data (>50%) were also excluded from the analysis.

The patients were divided into three main study groups: those who had recently been diagnosed with the rheumatic disease, those who developed the disease flare-up of previously diagnosed rheumatic disease, and those who had an infection during the follow-up of the rheumatic disease. Inflammatory arthritis, vasculitis, connective tissue diseases, and adult-onset Still disease (AOSD) were the rheumatic disease subgroups. An experienced rheumatologist reviewed the anamnesis, laboratory, and imaging data of the patients to confirm the newly diagnosed rheumatic diseases. Flare-up was defined as the need for newly prescribed immunosuppressive agents or increment of dose of the already prescribed immunosuppressive agents and NOT prescription of any antibiotics. Infection was defined as documentation of a possible infective agent via culture or imaging findings suggesting infection and prescription of any antibiotics. Patients with known rheumatic disease, coexisting malignancy, or any possible cause other than flare-up and infection (heart failure, etc.) were excluded.

### 2.2. Data assessment

Demographic (age at ESR ≥ 100 mm/h and sex) and laboratory data temporarily nearest to ESR ≥ 100 mm/h: complete blood count with differentials, aspartate aminotransferase (AST), alanine aminotransferase (ALT), creatinine, alkaline phosphatase (ALP), albumin, total protein, C-reactive protein (CRP), fibrinogen, ferritin, iron, total iron-binding capacity (TIBC), gamma-glutamyl transferase (GGT), mean red blood cell volume (MCV), red blood cell distribution width (RDW) and mean platelet volume (MPV), complement 3 (C3), complement 4 (C4), total cholesterol, triglyceride, high-density lipoprotein (HDL), low-density lipoprotein (LDL), and vitamin B12 levels were recorded.

### 2.3. Statistical analysis

IBM SPSS statistics version 25 (SPSS, Chicago, USA) was used for the statistical analysis. Categorical variables were expressed as numbers with percentages. Continuous data were presented as means (standard deviations) or medians and (minimum–maximum) based on their distribution. Chi-squared (post hoc Bonferroni correction) or Fisher’s exact tests, where appropriate, were used for comparison of groups for categorical variables. Intergroup differences were assessed by using one-way ANOVA (post hoc Tukey HSD) or the Kruskal–Wallis (post hoc Bonferroni) test for the continuous data. A decision tree by R (package party) was used to construct a clinician-friendly algorithm to discriminate between disease flare-ups and infection by using routine laboratory tests. Moreover, multivariable logistic regression analysis was done to find factors associated with infection over flare-up. Receiver operator curve (ROC) analysis was done to find cut-off values for relevant parameters, which can aid in differentiating rheumatic disease subgroups among patients with newly diagnosed rheumatic disorders. p < 0.05 was considered statistically significant.

## 3. Results

A total of 2442 individual patients who applied to our hospital between 2015 and 2020 and had ESR ≥ 100 mm/h were identified via the hospital database. Of these 2442 patients, 1257 were female (51.5%) and the median (min–max) age was 58.8 (3–97) years. Distribution of the main diagnostic groups was as follows: malignancy in 1061 (43.4%) patients [most common was lung cancer 136 (12.8%)], infection in 779 (31.9%) patients [most common was pneumonia 156 (20.0%)], and rheumatic disorders in 336 (13.8%) patients, and 266 (10.9%) patients did not have a definite diagnosis. As 25 of the 336 patients with rheumatic disorders had missing data, the remaining 311 patients constituted the main study group of these analyses. As the main focus of this study is the “rheumatic perspective”, no other result or discussion regarding the other diagnostic groups will be given.

### 3.1. Distribution of rheumatic diagnoses among main study groups

Of the 311 patients, 204 (65.5%) were female, with a mean age of 52.0 (17.5) (min–max: 18–90). Eighty-six (27.7%) patients had newly diagnosed rheumatic disease, 111 (35.7%) patients had rheumatic disease flare-up, and 114 (36.6%) patients had both rheumatic disease and infection. Inflammatory arthritis was the most common in the entire patient group (180 [57.9%]), followed by vasculitis (73 [23.5%]), connective tissue diseases (44 [14.1%]), and Still disease (14 [4.5%]) (Table 1). In individual disease perspective and irrespective of main study groups, the most common diseases were as follows: rheumatoid arthritis (96; 30.8%), spondyloarthritis (58; 18.7%), large vessel vasculitis (including PMR) (39; 12.5%), and systemic lupus erythematosus (22; 7.0%).

Vasculitis was the most common cause of ESR ≥ 100 mm/h in patients diagnosed with a new rheumatic disease (41; 47.7%), followed by inflammatory arthritis (27; 31.3%), connective tissue diseases (9; 10.5%), and adult Still disease (9; 10.5%) (Table 1). In disease flare-up group, inflammatory arthritis (92; 82.9%) [rheumatoid arthritis (56; 50.5%) and spondyloarthritis (30; 27.0%]) was the dominant diagnosis and other notable diseases include large vessel vasculitis (7; 6.3%) and SLE (5; 4.5%). In the infection group, rheumatoid arthritis and spondyloarthritis were the most common diseases, just as they were in the disease flare-up group. Another noteworthy point is that the study included a large number of patients (28/44 (63.6%)) with connective tissue disorders who had increased ESR due to infection during follow-up.**Evaluation of the laboratory parameters**

#### 3.1.1. Comparison of laboratory parameters between study groups (new diagnosis, flare-up, and infection)

When the laboratory parameters were compared at the group level, some differences existed among groups. Patients in the infection group had higher neutrophil counts, lower thrombocyte counts, higher MCV and MPV, higher transaminase levels, lower creatinine, lower albumin, and total protein levels, and higher CRP and B12 levels (Table 2). Patients with newly diagnosed rheumatic diseases had lower HDL and ferritin levels than those in the other groups.

**Figure 1:**
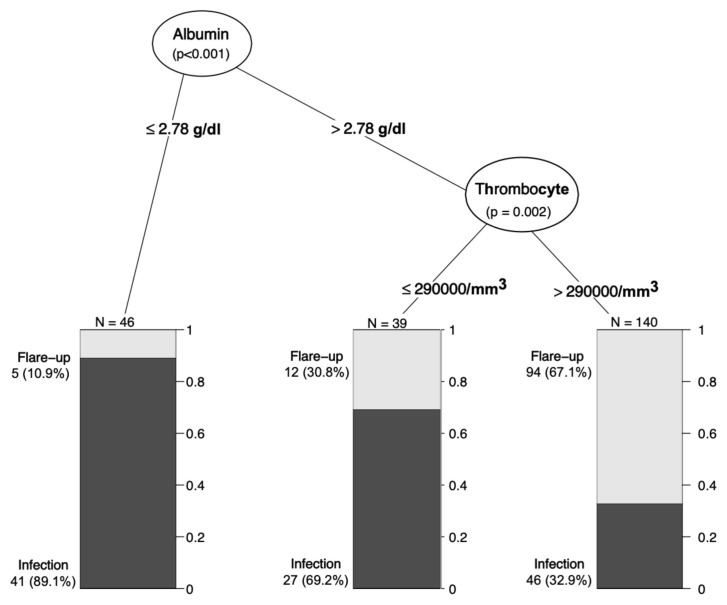
Decision tree to discriminate patients with disease flare-up and coexisting infection among patients with the rheumatic disease and ESR ≥ 100 mm/h.

To find variables that can be used to aid disease flare-up and infection discrimination, we have performed a decision-tree analysis (Figure 1). In this analysis, infection was found in 89.1% of the patients and flare-up was found in 10.9% when the serum albumin value was less than 2.78 g/dL. If the platelet count was less than 290,000/mm^3^, infection was present in 69.2% of patients with a serum albumin value greater than 2.78 g/dL, while flare-up was present in 30.8%. If the serum albumin level is greater than 2.78 g/dL and the platelet count is greater than 290,000/mm^3^, flare-up was diagnosed in 67.1% of patients, and infection was diagnosed in 32.9%. In multivariable analysis, these factors were associated with infection over flare-up (reference level): thrombocyte count (per 1000/mm^3^ increment) (aOR: 0.997 (0.995–0.999)), albumin (per 1 gr/dL increment) (aOR: 0.371 (0.218–0.630)), CRP (1 mg/dL increment) (aOR: 1.044 (1.011–1.044)).

#### 3.1.2. Comparison of laboratory parameters in each study group (new diagnosis, flare-up, and infection) according to rheumatic disease subgroups (inflammatory arthritis, vasculitis, connective tissue disorders, and adult Still disease)

**Figure 2:**
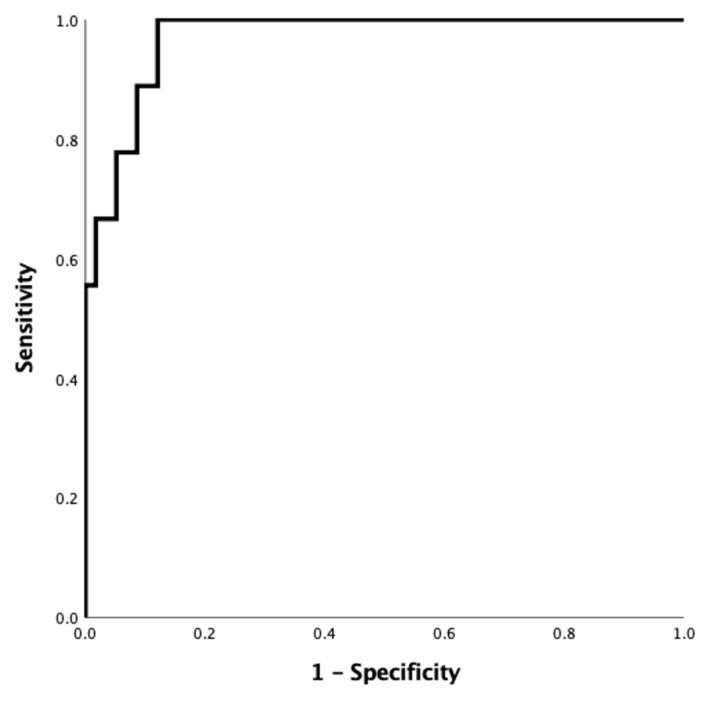
ROC analysis for ferritin to discriminate adult-onset Still disease from other rheumatic diseases in newly diagnosed rheumatic disease study group (AUC: 0.97 (0.93–0.99), p < 0.001).

Of patients with newly diagnosed rheumatic disease, patients with adult-Still disease had significantly higher ferritin levels: adult Still group 808 (357–15000 μg/mL), inflammatory arthritis 105 (14–795), vasculitis 171 (3–653), and connective tissue diseases 154 (3–497) (p < 0.001) (Table 3). The ROC curve analysis revealed that it had an 89% sensitivity and 92% specificity for distinguishing adult-onset Still disease from other rheumatic diseases with the cut-off value of 440 μg/mL for ferritin (Figure 2) (AUC: 0.97 (0.93–0.99), p < 0.001).

Among patients with disease flare-ups, complement three levels were significantly lower in patients with connective tissue disorders compared to inflammatory arthritis and vasculitis groups (Table 4). Lastly, of patients in the main infection group, patients with connective tissue disorders were younger, had a higher likelihood of female sex, and had higher MPV and RDW levels. Albumin levels in patients with vasculitis were significantly lower than in inflammatory arthritis and connective tissue disorders groups; also, ALT, fibrinogen, and ferritin levels were higher in patients with vasculitis than in these two groups (Table 5).

## 4. Discussion

The present study evaluated 2442 patients with an ESR ≥ 100 mm/h. Malignancy was the most common etiological cause, accounting for 43.4% of all cases, followed by infection (31.9%) and rheumatic diseases (13.8%). Patients with rheumatic diseases can be divided into three main study groups: new diagnosis, rheumatic disease flare-up, and infections added to rheumatic disease. Vasculitis was the most common cause in newly diagnosed patients, while inflammatory arthritis was prominent in patients with disease flare-ups. Inflammatory arthritis was in the foreground in patients with rheumatic disease and infection, while connective tissue diseases were another critical group of patients in this regard. We hypothesized that a diagnostic algorithm consisting of serum albumin and platelets could be helpful in differentiating rheumatic disease flare-ups and coexisting infections, which is an important clinical problem.

Similar to the fever of unknown origin, patients with ESR ≥ 100 mm/h fall into one of the three disease groups: malignancies, infections, and inflammatory rheumatic diseases. In our study, unlike the literature, malignancy was encountered more frequently than infectious diseases among the etiological causes. In the study of Otero-Castro et al., which included 879 patients between 2002 and 2014, infections were in the first line with 42%, malignancy in the second line with 22%, and autoimmune-inflammatory diseases in the third line with 13% [6]. In the study by Lluberas-Acosta and Schumacher in hospitalized patients with an ESR ≥ 100 mm/h, infections were the most common etiological cause and rheumatic diseases the second [7]. While malignancies and infectious diseases were determined in the foreground in our study, since our center is a reference center that serves especially oncological patients in Turkey, inflammatory rheumatic diseases occur significantly.

The literature generally provides a distribution of patients with an ESR ≥ 100 mm/h. In our study, unlike the literature, we proposed a new grouping perspective for patients with rheumatic disease and ESR ≥ 100 mm/h, which we believe is more appropriate for clinical settings. In daily practice, these patients present with newly diagnosed rheumatic diseases, rheumatic disease flare-ups, and rheumatic disease with infections. In line with the prevalence of the disorders in general population, inflammatory arthritides (mainly rheumatoid arthritis (RA) and spondyloarthritis (SpA)) were the most common diseases among all patients. In a recent study by Daniels et al. from the Mayo clinic, RA was found to be the prominent disease [4]. On the other hand, vasculitis and related diseases came to the fore in the newly diagnosed rheumatic diseases group. In this respect, large vessel vasculitis and, less frequently, ANCA-associated vasculitis were the diseases that should always be kept in mind. Inflammatory arthritis was the most prominent cause, especially in the group of patients with rheumatic disease flare-ups, approximately 83% of all exacerbations were associated with inflammatory arthritis, especially RA.

Two conditions are considered in the foreground if a patient with known rheumatic disease is admitted with ESR ≥ 100 mm/h. The first is a flare-up of the existing disease, and the second is the coexisting infections, possibly due to the immunosuppressive drugs used. It is important to distinguish between these two conditions because the treatment approaches are entirely different. As the prevalence of the diseases is higher, we found more RA and SpA patients with infections; however, infections were also common in patients with systemic lupus erythematosus and ANCA-associated vasculitis. Even though connective tissue disorders are relatively uncommon conditions, it should be noted that they can be complicated by infections and result in an ESR ≥ 100 mm/h. On the other hand, we have also investigated whether simple laboratory tests can be directive in daily practice. From this point of view, two laboratory tests come to the fore in patients with ESR ≥ 100 mm/h: albumin and thrombocyte count. Albumin is a well-known negative acute phase reactant. A mean value of 3.3 g/dL was observed in newly diagnosed rheumatic diseases. Although this value is lower than normal, it corresponds to a mild hypoalbuminemia. In the rheumatic disease flare-up group, albumin levels were at the lower limit of normal (mean 3.6 g/dL). On the other hand, when the infection is added to the rheumatic disease, the mean albumin levels reach a lower level than the other groups. In the decision-tree analysis, it was seen that albumin could be very valuable in differentiating exacerbation and infection, with the cut-off value of albumin as 2.78 g/dL. In addition, thrombocytosis was more prominent in the flare-up group, while it was less frequent in infectious diseases (66% vs. 44%). With all these results, a decrease in albumin below 2.78 g/dL should suggest infections rather than flare in the patient group with ESR >100. In the presence of albumin levels above 2.78 g/dL, a platelet count of 290,000/mm^3^ and above supports flare-up. This diagnostic algorithm may be simple and instructive for the clinician but should be supported by other studies.

Adult Still disease was considered a separate rheumatic disease subgroup in the study as the disease has unique features. Especially among newly diagnosed rheumatic diseases, AOSD has a prominent seat. One of the points that make AOSD different is the high ferritin levels, which are more prominent than in other groups. As a matter of fact, in a previous study by us, it was determined that a serum ferritin level more than five times of the normal level with arthralgia is highly indicative of adult Still disease [8]. Similarly, Ushiyama et al. reported that serum ferritin levels higher than five times the normal levels have a sensitivity of 74.8% and a specificity of 83.2% for adult Still disease [9]. In the light of this information, it can be said that the high ferritin level in the patient group with ESR > 100 should put adult Still disease in the foreground.

The most important limitation of our study is that it was a chart review. All possible inborn biases of this kind of study can be applied to our research. On the other hand, our study has several strengths. We followed a strict inclusion-exclusion strategy. Moreover, our study contains valuable data as it can lead to prospective long-term follow-up studies.

In conclusion, high ESR still maintains its value in the diagnostic process. Among patients with ESR ≥ 100 mm/h, rheumatic diseases are the most critical etiologic causes besides malignancy and infection. Coexisting infection was the most common cause of ESR ≥ 100 mm/h in patients with rheumatic diseases, followed by disease flare-ups. When biochemical parameters were evaluated, serum ferritin levels in newly diagnosed rheumatic diseases indicated adult-onset Still disease. Serum albumin levels and thrombocyte counts may play a role in discriminating rheumatic disease flare-ups from coexisting infections.

## Figures and Tables

**Table 1:** Distribution of patients with ESH ≥ 100 using rheumatic disease subgroups and individual rheumatic diagnoses grouped according to main study group (N = 311).

			Main study groups			
		All patients, N = 311	Newly diagnosed rheumatic diseases, N = 86 (27.7%)	Rheumatic disease activation, N = 111 (35.7%)	Infection in rheumatic diseases, N = 114 (%36,6)	p-value
**Female, N (%)**		204 (65.5)	56 (65.1)	85 (76.6)	63 (55.3)	0.003*
**Age, (mean (SD)), years**		52.0 (17.5)	53.4 (17.7)	48.1 (17.5)	54.5 (16.7)	0.014**
**Rheumatic** **disease subgroups**						<0.001♣
**Inflammatory arthritis**		180 (57.9)	27 (31.3)	92 (82.9)	61 (53.5)	<0.001*
	Rheumatoid arthritis	96 (30.9)	13 (15.1)	56 (50.5)	27 (23.7)	
	Gout-crystal arthritis	13 (4.2)	5 (5.8)	2(1.8)	6 (5.3)	
	Spondyloarthritis	58 (18.7)	8 (9.3)	30 (27.0)	20 (17.5)	
	JIA	4 (1.3)	0	2 (1.8)	2 (1.8)	
	FMF	9 (2.9)	1 (1.2)	2 (1.8)	6 (5.3)	
**Vasculitis & assoc. dis**.		73 (23.5)	41 (47.7)	11 (9.9)	21 (18.4)	<0.001*
	GVV & PMR	39 (12.5)	25 (29.1)	7 (6.3)	7 (6.1)	
	AAV	18 (5.8)	7 (8.1)	2 (1.8)	9 (7.9)	
	SMV	3 (1.0)	0	0	3 (2.7)	
	Polyarteritis nodosa	3 (1.0)	2 (2.3)	1 (0.9)	0	
	Behcet’s syndrome	3 (1.0)	1 (1.2)	1 (0.9)	1 (0.9)	
	Rheumatoid vasculitis	1 (0.3)	1 (1.2)	0	0	
	IgG4-related disease	5 (1.6)	5 (5.8)	0	0	
	Cerebral vasculitis	1 (0.3)	0	0	1 (0.9)	
**Connective tissue dis**.		44 (14.1)	9 (10.5)	7 (6.3)	28 (24.5)	<0.001*
	SLE	22 (7.0)	5 (5.8)	5 (4.5)	12 (10.5)	
	Systemic sclerosis	7 (2.2)	1 (1.2)	1 (0.9)	5 (4.4)	
	Sarcoidosis	5 (1.6)	2 (2.3)	0	3 (2.6)	
	Sjogren’s syndrome	3 (1.0)	0	0	3 (2.6)	
	Myositis	2 (0.6)	0	0	2 (1.8)	
	Overlap syndrome	1 (0.3)	0	1 (0.9)	0	
	Relapsing polychondritis	2 (0.6)	1 (1.2)	0	1 (0.9)	
	Retroperitoneal fibrosis	1 (0.3)	0	0	1 (0.9)	
	Granulomatous mastitis	1 (0.3)	0	0	1 (0.9)	
**Adult still disease**		14 (4.5)	9 (10.5)	1 (0.9)	4 (3.5)	0.03*

FMF: Familial mediterranean fever, AAV: ANCA-associated vasculitis, GVV: Great vessel vasculitis, JİA: Juvenile idiopathic arthritis, SMV: Small vessel vasculitis, PMR: Polymyalgia rheumatica, SLE: Systemic lupus erythematosus.

Percentages are column percentages

♣ Corresponds to p-value of comparison of disease subgroups among main study groups

* p-value of chi-squared test from the comparison of each rheumatic disease subgroup frequencies between main study groups

Post hoc comparison for the source of difference (via Bonferroni correction):

Inflammatory arthritis: Rheumatic disease activation

Vasculitis & assoc. dis: Newly diagnosed rheumatic disorders

Connective tissue disorder: Infection in rheumatic diseases

Adult Still disease: Newly diagnosed rheumatic disorders

** p-value of one way analysis of variances. Difference was caused by rheumatic disease activation group (post hoc Tukey HSD).

**Table 2:** Comparison of laboratory values between main study groups.

	Main study groups						
Variable	Newly diagnosed rheumatic diseases (n = 86)		Rheumatic disease activation (n = 111)		Infection in rheumatic diseases (n = 114)		P† value
**Hemoglobin (mg/dL)**	10.1 (1.5)		10.2 (1.3)		10.0 (1.5)		0.74
**Anemia (<12)**	80 (93.0)		101 (91.8)		102 (89.5)		0.66
**Leukocyte (/mm** **^6^** **)**	9.8 (3.3)		9.4 (3.9)		10.3 (4.3)		0.17
**Leukocytosis (≥10,000)**	35 (40.7)		37 (33.3)		55 (48.3)		0.07
**Neutrophil(/mm** **^6^** **)**	6.9 (2.9)		6.5 (3.7)		7.8 (4.1)		**0.036**
**Neutrophilia (≥6400)**	43 (50.0)		45 (41.3)		66 (57.9)		**0.046**
**Thrombocyte(/mm** **^6^** **)**	432 (168)		441 (178)		350 (152)		**<0.001**
**Thrombocytosis (≥350)**	60 (69.8)		73 (66.4)		50 (43.9)		**<0.001**
**MCV**	77.6 (7.8)		75.7 (9.5)		81.6 (8.9)		**<0.001**
**RDW**	17.2 (3.5)		17.5 (2.7)		17.2 (3.0)		0.67
**MPV**	7.6 (0.9)		7.6 (0.9)		8.0 (1.0)		**0.004**
	**N**		**N**		**N**		
**AST (IU)**	85	17 (7–107)	108	19 (9–125)	114	22.9 (8–133)	**0.01**
**ALT (IU)**	86	17 (3–165)	110	14 (5–230)	114	18 (4–232)	0.08
**ALP (IU)**	75	98 (45–658)	77	90 (34–335)	108	98.5 (35–746)	0.16
**GGT (IU)**	74	34.5 (11–485)	76	25 (2–525)	108	40.5 (9–388)	**0.001**
**Creatinine (mg/dL)**	85	0.7 (0.4–10)	108	0.7 (0.3–5.4)	114	0.7 (0.3–9.1)	**0.02**
**Albumin (g/dL)**	84	3.3 (0.5)	108	3.6 (0.6)	114	3.1 (0.7)	**<0.001**
**Total Protein (g/dL)**	83	7.0 (0.7)	107	7.2 (0.9)	114	6.7 (1.2)	**<0.001**
**CRP (mg/dL)**	86	10(0.3–32)	110	8 (0.2–45)	114	13 (0.5–51)	**<0.001**
**Fibrinogen (mg/dL)**	31	579 (151)	34	486 (187)	60	538 (189)	0.17
**HDL**	59	42.3 (17.0)	82	50.5 (14.4)	76	44.4 (19.2)	**0.01**
**Triglyceride**	59	118 (59–408)	84	110 (44–858)	82	138 (42–740)	0.07
**B12**	64	254 (74–1500)	85	245 (79–1500)	96	296(98–1500)	**0.05**
**C3**	45	140.0 (45.6)	25	128.1 (39.8)	56	124.1 (35.9)	0.14
**C4**	44	28.8 (14.6)	23	23.1 (8.2)	56	26.0 (10.7)	0.17
**Ferritin (μg/mL)** *	67	170 (3–15000)	77	31 (3–588)	97	167 (8–8597)	**<0.001**

AST: Aspartate aminotransferase, ALT: Alanine aminotransferase, ALP: Alkaline phosphatase, CRP: C-reactive protein, GGT: Gamma-glutamyl transferase, MCV: Mean corpuscular volume, MPV: Mean platelet volume, RDW: Red Cell Distribution Width.

† p-value calculated with one way ANOVA (post hoc Tukey HSD), if the variable was described by using mean (standard deviation) (with chi-squared test (post hoc Bonferroni correction) if the variable was described using by n(%), with the Kruskal–Wallis (post hoc Bonferroni HSD) test if the variable was described by using median (minimum–maximum).

As a result of post hoc tests (Tukey HSD or Bonferroni) in the variables in which a statistically significant difference was detected between the groups, the groups that caused the difference were:
**Group 1 vs. group 2:** HDL **Group 1 vs. group 3:** AST **Group 2 vs. group 3:** neutrophil count, creatinine, GGT **Group 2 vs group 1 and 3:** albumin, ferritin **Group 3 vs group 1 and 2**: thrombocyte count, MCV, MPV, total protein, CRP, B12

Data were given as mean (standard deviation) or n(%), if otherwise specified.

* median (minimum–maximum).

**Table 3:** Comparison of laboratory tests according to rheumatic disease subgroups* in patients newly diagnosed with rheumatic disease*.

Variable	Inflammatory arthritis n = 27		Vasculitis n = 41		Connective tissue disorders n = 9		Adult Still disease n = 9		p*
**Female, n(%)**	17 (63.0)		28 (68.3)		5 (55.6)		6 (66.7)		0.89**
**Age**	58 (20–80)		59 (18–77)		56 (32–79)		52 (19–69)		0.68
**Hemoglobin (mg/dL)**	9.9 (7.2–15.6)		9.9 (7.3–15.3)		9.5 (8.1–11.9)		10.1 (7.5–11.7)		0.74
**Leukocyte (/mm** **^6^** **)**	9.1 (4.7–18.6)		9.7 (5.1–18.3)		8.6 (4.3–19.4)		9.0 (5.6–13.3)		0.66
**Neutrophil (/mm** **^6^** **)**	6.9 (2.1–16.1)		6.2 (3.1–14.7)		5.6 (2.8–9.1)		7.4 (3.0–10.3)		0.45
**Thrombocyte(/mm** **^6^** **)**	466 (157–1248)		442 (166–882)		301 (245–531)		372 (110–639)		0.09
**MCV**	80.0 (58.1–89.5)		78.5 (62.6–88.3)		77.2 (62.1–91.4)		78.2 (57.2–87.5)		0.99
**RDW**	16.3 (12.7–26.8)		17.0 (13.1–32.2)		17.4 (14.1–27.8)		16.8 (7.2–22.7)		0.64
**MPV**	7.4 (6.3–9.2)		7.8 (5.0–9.3)		8.2 (6.2–9.3)		7.8 (6.7–8.9)		0.78
	***N***		***N***		***N***		***N***		
**AST (IU)**	26	17.5 (7.0–107.0)	41	16.0 (8.0–50.0)	9	16.0 (10.0–47.0)	9	44.0 (20.0–63.0)	0.08
**ALT (IU)**	27	16.0 (5.0–60.0)	41	17.0 (3.0–80.0)	9	19.0 (5.0–37.0)	9	28.0 (8.0–165.0)	0.26
**ALP (IU)**	20	97.0 (45.0–658.0)	39	104.0 (63.0–354.0)	8	66.0 (59.0–272.0)	8	134.0 (61.0–326.0)	0.26
**GGT (IU)**	21	45.0 (11.0–383.0)	37	35.0 (11.0–206.0)	8	15.5 (12.0–211.0)	8	53.0 (16.0–485.0)	0.29
**Creatinine (mg/dL)**	27	0.6 (0.4–1.4)	41	0.7 (0.4–10.4)	8	0.6 (0.4–2.8)	9	0.7 (0.5–0.8)	0.32
**Albumin (g/dL)**	27	3.3 (2.4–4.7)	40	3.2 (1.0–4.4)	9	3.3 (3.0–3.9)	8	3.2 (2.8–4.2)	0.76
**Total protein (g/dL)**	26	7.0 (5.3–8.2)	49	7.2 (5.4–8.4)	9	6.8 (5.7–8.7)	8	7.3 (6.1–8.1)	0.48
**CRP (mg/dL)**	27	10.4 (1.5–31.0)	41	10.7 (1.6–32.0)	9	8.7 (0.3–17.7)	9	8.4 (4.3–26.0)	0.43
**Fibrinogen (mg/dL)**	6	581 (225–759)	14	629 (270–891)	5	616 (476–793)	6	523 (390–616)	0.47
**HDL**	18	40 (22–64)	26	43 (20–113)	6	44 (30–56)	9	29 (20–60)	0.18
**Triglyceride**	18	103 (59–408)	26	118 (62–272)	7	141 (70–335)	8	180 (84–265)	0.39
**B12**	21	266 (109–1081)	41	235 (74–717)	9	266 (162–1500)	3	252 (187–571)	0.88
**C3**	8	148 (107–237)	24	150 (38–222)	6	90 (42–152)	7	167 (76–216)	0.09
**C4**	8	32 (5–43)	24	29 (2–67)	6	19 (7–39)	6	27 (10–69)	0.45
**Ferritin (** **μ** **g/mL)**	17	105 (14–795)	33	171 (3–653)	8	154 (3–497)	9	808 (357–15000)	**<0.001**

Data were given as median (minimum–maximum)) or n(%), if otherwise specified

* The Kruskal–Wallis test,

** Chi-squared test.

**Table 4:** Comparison of laboratory tests according to rheumatic disease subgroups in patients presenting with flare-up of rheumatic disease (The adult Still group was not included in the analysis because of only 1 patient).

Variable	Inflammatory arthritis n = 92		Vasculitis n = 11		Connective tissue disorders n = 7		p*
**Female, n(%)**	70 (76.1)		8 (72.7)		8 (85.7)		0.81**
**Age**	49 (18–90)		33 (19–79)		42 (21–65)		0.42
**Hemoglobin (mg/dL)**	10.1 (7.1–14.4)		9.4 (7.3–12.0)		10.6 (9.6–11.9)		0.09
**Leukocyte (/mm** **^6^** **)**	8.8 (3.8–31.0)		8.2 (4.5–14.0)		8.3 (5.3–10.5)		0.49
**Neutrophil (/mm** **^6^** **)**	5.7 (0.7–27.6)		6.3 (2.3–9.9)		6.3 (3.0–8.9)		0.88
**Thrombocyte (/mm** **^6^** **)**	402 (78–1228)		418 (311–904)		327 (291–649)		0.36
**MCV**	76.3 (57.3–96.1)		78.5 (59.0–96.3)		72.6 (58.0–79.3)		0.24
**RDW**	17.1 (11.0–28.5)		17.1 (14.0–20.8)		17.4 (14.0–22.6)		0.92
**MPV**	7.6 (5.8–9.9)		7.6 (6.4–10.7)		8.0 (7.4–9.2)		0.18
	**N**		**N**		**N**		
**AST (IU)**	89	19.0 (9.0–125.0)	11	16.0 (10.0–60.0)	7	24.0 (9.0–76.0)	0.23
**ALT (IU)**	91	14.0 (5.0–230.0)	11	11.0 (6.0–51.0)	7	16.0 (11.0–38.0)	0.41
**ALP (IU)**	61	93 (24–274)	10	89 (51–335)	6	87 (50–175)	0.91
**GGT (IU)**	60	25.5 (2.0–525.0)	10	20.0 (9.0–275.0)	6	22.5 (10.0–69.0)	0.76
**Creatinine (mg/dL)**	89	0.7 (0.3–5.4)	11	0.7 (0.4–0.9)	7	0.6 (0.5–0.8)	0.98
**Albumin (g/dL)**	89	3.6 (2.5–7.0)	11	3.5 (2.6–4.1)	7	3.5 (3.0–3.9)	0.44
**Total protein (g/dL)**	88	7.3 (5.9–9.3)	11	6.9 (5.6–8.3)	7	7.4 (6.5–8.0)	0.26
**CRP (mg/dL)**	91	8.1 (0.2–45.4)	11	9.2 (0.4–34.4)	7	3.0 (0.2–24.0)	0.30
**Fibrinogen (mg/dL)**	26	489 (182–910)	6	560 (296–747)	1	241	0.30
**HDL**	68	51 (22–91)	10	46 (18–70)	4	61 (45–65)	0.27
**Triglyceride**	70	113 (44–363)	10	80 (50–858)	4	168 (88–221)	0.13
**B12**	70	246 (87–1500)	10	250 (158–384)	5	243 (79–746)	0.93
**C3**	15	136 (69–209)	4	166 (116–191)	6	84 (61–130)	**0.032**
**C4**	13	23 (17–44)	4	23 (19–31)	6	16 (8–28)	0.29
**Ferritin (** **μ** **g/mL)**	61	31 (3–588)	9	28 (8–285)	6	23 (9–160)	0.86

Data were given as median (minimum–maximum)) or n(%), if otherwise specified.

* The Kruskal–Wallis test,

** Chi-squares test.

**Table 5:** Comparison of laboratory tests according to rheumatic disease subgroups in patients presenting with infection (The adult Still group was not included because of only 1 patient).

Variable	Inflammatory arthritis n = 61		Vasculitis n = 21		Connective tissue disorders n = 28		p*
**Female, n(%)**	33 (54.1)		5 (23.8)		22 (78.6)		0.01**
**Age**	59 (19–88)		62 (33–82)		48 (18–77)		0.03
**Hemoglobin (mg/dL)**	10.0 (7.1–13.9)		10.0 (7.0–12.1)		9.8 (7.5–12.4)		0.60
**Leukocyte (/mm** **^6^** **)**	10.8 (1.6–23.0)		10.1 (2.8–25.7)		8.6 (0.1–17.7)		0.19
**Neutrophil (/mm** **^6^** **)**	8.0 (0–21.0)		7.5 (1.3–23.3)		6.0 (0–14.1)		0.26
**Thrombocyte (/mm** **^6^** **)**	345 (114–878)		323 (134–876)		330 (72–703)		0.27
**MCV**	84.1 (58.2–101.0)		83.0 (67.9–95.8)		77.3 (61.9–94.8)		0.05
**RDW**	16.6 (13.3–25.6)		15.6 (13.1–33.6)		17.5 (13.7–26.6)		0.028
**MPV**	7.9 (5.9–10.0)		7.4 (5.8–9.0)		8.3 (6.4–10.8)		0.018
	N		N		N		
**AST (IU)**	61	20.0 (8.0–133.0)	21	26.0 (9.0–110.0)	28	19.5 (10.0–106.0)	0.28
**ALT (IU)**	61	15.0 (4.0–139.0)	21	28.0 (7.0–232.0)	28	18.5 (4.0–67.0)	0.023
**ALP (IU)**	56	97 (35–440)	21	103 (60–325)	27	92 (48–746)	0.61
**GGT (IU)**	56	39 (10–195)	21	57 (17–320)	27	38 (9–388)	0.16
**Creatinine (mg/dL)**	61	0.7 (0.3–8.2)	21	0.9 (0.4–9.2)	28	0.6 (0.4–8.3)	0.08
**Albumin (g/dL)**	61	3.2 (1.3–5.3)	21	2.9 (2.1–3.8)	28	3.3 (1.8–4.7)	0.032
**Total protein (g/dL)**	61	6.9 (3.5–9.1)	21	6.7 (5.2–8.1)	28	6.5 (5.2–8.6)	0.61
**CRP (mg/dL)**	61	13.5 (0.6–46.0)	21	15.0 (3.8–37.1)	28	12.5 (0.5–50.6)	0.30
**Fibrinogen (mg/dL)**	32	486 (250–944)	13	561 (501–930)	13	516 (286–638)	0.036
**HDL**	39	43 (17–87)	14	32 (17–83)	22	37 (19–93)	0.59
**Triglyceride**	43	129 (42–382)	14	134 (70–585)	24	153 (67–740)	0.14
**B12**	51	289 (98–1500)	18	339 (104–916)	25	265 (110–949)	0.66
**C3**	20	125 (48–216)	11	136 (99–156)	22	122 (41–209)	0.70
**C4**	20	28 (3–38)	11	31 (17–53)	22	19 (10–47)	0.17
**Ferritin (** **μ** **g/mL)**	48	159 (14–2334)	19	355 (25–3010)	26	106 (8–3020)	0.015

Data were given as median (minimum–maximum)) or n(%), if otherwise specified.

* The Kruskal–Wallis test,

** Chi-squared test.

## Data Availability

Data are available upon request from the corresponding author.
